# Aqueous extract of post-fermented tea reverts the hepatic steatosis of hyperlipidemia rat by regulating the lipogenic genes expression and hepatic fatty acid composition

**DOI:** 10.1186/1472-6882-14-263

**Published:** 2014-07-23

**Authors:** Jie Zhou, Liang Zhang, Jingsong Zhang, Xiaochun Wan

**Affiliations:** Key Laboratory of Tea Biochemistry & Biotechnology, Ministry of Education and Ministry of Agriculture, Anhui Agricultural University, Hefei, 230036 China

**Keywords:** Post-fermented tea, Lipids, Absorption, Lipogenic genes, Leptin

## Abstract

**Background:**

Post-fermented tea has been used for the prevention of metabolic syndrome in Western China. Present study reports the biochemical mechanism of lipid-lowering effects of Jing-wei fu tea (JWFT), a variety of post-fermented tea on high-fat diet-induced hyperglycemia and obesity in rats.

**Methods:**

Aqueous extract of JWFT was prepared by putting them in boiling water, and then concentrated under reduced pressure. The major compounds of JWFT were determined by high performance liquid chromatography (HPLC). High-fat diet fed rats were orally administered different doses of JWFT aqueous extract (0.5, 1.0 and 2.0 g/kg) for four weeks. At the end of this experiment, hepatic lipids, serum leptin and lipids levels were determined using enzyme-linked immunosorbent assay (ELISA). The hepatic fatty acid composition was analyzed using gas chromatography mass (GC-MS). The relative expression of lipids metabolism genes was analyzed using reverse transcriptase-polymerase chain reaction (RT-PCR).

**Results:**

The results showed that JWFT inhibited the increase in the body weight, abdominal adipose weight, serum lipids and hepatic lipids, and decreased serum leptin levels of high-fat diet fed rats. JWFT normalized hepatic fatty acid composition of hyperlipidemia rats by up-regulating hepatic acetyl-CoA carboxylase (*ACC*), stearoyl-CoA desaturase 1 (*SCD1*) and fatty acid synthase (*FAS*) gene expression, and down-regulating carnitine palmitoyl transferase-1 (*CPT1*). Furthermore, the results showed that JWFT inhibited the absorption of lipids.

**Conclusion:**

JWFT could mitigate the obesity-induced hepatic steatosis by regulating hepatic lipogenesis and lipolysis.

## Background

Obesity is a balance disruption of energy intake and expenditure [1]. As a typical symptom of metabolic syndrome, hepatic steatosis was usually induced by high-fat and high carbohydrate diet [2]. Liver is the main organ for lipid uptake, de novo synthesis and fatty acid lipolysis [3]. Some lipogensis and lipolysis enzymes have been known to be involved in the lipids synthesis and metabolism. For example, acetyl-CoA carboxylase (*ACC*) converts the acetyl-CoA to malonyl-CoA, which is the direct carbon donor for de novo synthesis of fatty acid. Fatty acid synthase (*FAS*) organizes the structural skeleton of fatty acid with malonyl-CoA. Stearoyl-CoA desaturase 1 (*SCD1*) is a critical rate-limiting enzyme of de novo synthesis of monounsaturated fatty acids (MUFAs) [4]. Furthermore, increase in the carnitine palmitoyltransferase 1 (*CPT1*) substantially reduced the hepatic triglycerides level because it increased the fatty acid oxidation [5]. While the deficiency of SCD1 increased the oxidation of fatty acid, insulin sensitivity and resistance to diet-induced obesity and liver steatosis [6].

Many traditional herbal materials have attracted much attention because their various health benefits [7, 8]. There has been a very long history of tea consumption as beverage or herbal medicine. Tea contains high contents of polyphenols and caffeine. Its biological activities have been widely studied with respect to cancer prevention, anti-inflammatory, anti-hyperlipidemia and antimicrobial effects [9, 10]. Recently, post-fermented teas including pu-erh tea and fuzhuan brick tea have been reported with beneficial efficacy for metabolic syndrome in clinical intervention trials and animal models [11]. For many years, catechins and their gallate esters were widely reported as the main active compounds of green tea for anti-cancer and anti-obesity. In addition, (−)-epigallocatechin gallate (EGCG) showed the effects of inhibiting lipids absorption and synthesis [12]. But after post-fermentation, these compounds were highly decreased compared with unfermented tea. Therefore, it was suggested the catechins’ metabolite formed in post-fermentation may contribute to the health benefits of post-fermented tea [13].

“Fu” tea is a kind of post-fermented tea according to the classification of processing method. Its history could be dated back to the Ming Dynasty during 1500 A.D. [14]. Jing-wei fu tea (JWFT) is made of the mature leaves of *Camellia sinensis* by undergoing an intensified fermentation of *Eurotium cristatum* [15]. The detailed manufacture process of this tea could be described as panning, rapid pile fermentation, rolling, drying, softening with steam, piling, tea brick pressing, fungal fermentation, and drying in order. The phase of “fungal fermentation” is critical for typical aroma, taste and unique phytochemical profiles.

The aim of this paper is to elucidate JWFT’s function of regulating lipid metabolism and provide a novel understanding on lipid-lowering and anti-obesity activities of tea.

## Methods

### Materials

The raw material of Jing-wei fu tea is the mature large leaves of *Camellia sinensis* L. O. Kuntze. Jing-wei fu tea (JWFT) was provided by Cang-shan Co, Ltd. (Xianyang, Shaanxi, China) and formally identified by Professor Xiaochun Wan. Tea leaves were collected from tea plants (*Camellia sinensis*) in the Hanzhong district of China. To process tea, the mature leaves were dampened and fermented with the *Eurotuim cristotium* under high humidity and temperature. All of the chemical standards used in this study were with the purity of more than 98%.

### Preparation and analysis of JWFT extract

Aqueous extract was prepared by boiling minced JWFT powder with distilled water for 40 min at a ratio of 1:5 (w/v) three times. The filtered solution was combined and concentrated at 60°C with rotary evaporator to the required concentration, and then stored at 4°C. The procedures of sample preparation and HPLC analysis referenced published methods [16].

### Animals and treatment

Fifty male Sprague–Dawley (SD, 16 weeks old) rats were purchased from the Animal Breeding and research center of Anhui Medical University, China. This study was approved by the Institutional Animal Care and Use Committee of Anhui agricultural university and carried out according to the guidelines of Care and Use of Animals Laboratory. These rats were housed in an environmentally controlled room (22 ± 2°C, 60 ± 5% relative humidity, and 12 hours light/dark cycle) and received food and water *ad libitum*. At the beginning of study, the body weights of rats were ranged from 160 to 200 g. After one week’s acclimatization, rats were randomly divided into five groups of 10 animals each. Four groups were fed high-fat diet and the fifth group was fed normal control diet (NC). One group of high-fat diet fed rats served as control group (HFC) and was maintained on the high-fat diet alone until the end of the study, while other three groups received high-fat diet and daily oral administrations (20 ml/kg) of JWFT extract for four weeks, at the dose of 0.5, 1.0 and 2.0 g/kg body weight, named as low (LJT), medium (MJT) and high (HJT) dose of JWFT treatment group. After four weeks’ treatment, the animals were fasted for 12 hours, and then blood was taken from the femoral artery into tubes and immediately centrifuged at 6000 rpm at 4°C for 5 min. All the samples were stored at −80°C until analyzed.

### Body weight, abdominal fat, serum lipids and leptin analysis

Each rat’s body weight was recorded every week during the whole experiment. At the last day, rats were fasted for 12 h but free access to water, and then were anaesthetized. Blood was drawn from the abdominal aorta and rats were killed by exsanguinations. The intra-abdominal fat (including epididymal, mesenteric, and retroperitoneal fat) were removed and weighed. The body weights and abdominal fat of treatment groups were statistically compared with the HFC group. The levels of cholesterol, triglycerides and high density lipoprotein-cholesterol (HDL-C) were measured using ELISA methods with an automatic analyzer (SPECTRA MAX 190, Molecular Devices, US). Low density lipoprotein-cholesterol (LDL-C) levels were determined using the formula: LDL-C = TC-(1/5TG + HDL-C) according to the published methods [11].

The levels of serum leptin were determined using automatic radioimmunoanalyzer according to the leptin radioimmunoassy kit manufacturer’s (Beijing Northern institute of biotechnology, Beijing, China) directions.

### Extraction and measurement of hepatic lipids

Total liver lipid was extracted by homogenizing with chloroform-methanol mixture (2:1, v/v) to a final dilution 20-fold the volume of the tissue sample according to the published method [17]. The extraction was carried out in a tissue tearor for three times. Pooled extract was dried using a nitrogen evaporator. Hepatic total cholesterol (TC) and triglycerides (TG) were determined by ESLIA according to the manufacturer’s directions.

### Preparation and analysis of fatty acid methyl ester

Fatty acid methyl ester (FAME) was prepared by a safe rapid mean for methyl esterification with potassium hydroxide-methanol as a reaction solution, and then was extracted with hexane. 200 mg of lipids dissolved in 2 mL hexane and mixed well, then added 0.2 mL KOH-methanol solution (2 M). These tubes were mixed by vortex agitation for 30 sec and centrifuged at 5000 rpm for 5 min. The supernatant containing FAME was decanted and diluted with hexane, and then added 2 g sodium sulfate anhydrous for overnight.

Gas chromatograph-mass spectrometer (GC-MS) analysis of fatty acids composition was performed using Shimadzu GC-MSQP 2010S system (Tokyo, Japan) equipped with a DB-5 capillary column (30 m × 0.32 mm ID) (Agilent, USA). The injection volume was 1 μL and split less. Helium was used as carrier gas and flow rate at 0.85 mL/min. The oven temperature program was from 150°C to 190°C at 18°C/min maintained for 1 min, 190°C to 200°C at 0.5°C/min held for 5 min, 200°C to 230°C at 2°C/min kept for 5 min. The injection and detector (FID) temperatures were 250°C and 280°C respectively. Mass detection was done with electric ionization at 1 keV by using selected-ion monitoring/scan mode for masses in the range of *m*/*z* at 50 to 600.

### Analysis of the fecal lipids

In the 27th day, tea treatment and high-fat diet control groups’ animals were fasted for 12 h. After that, tea treatment groups were orally administered corresponding dose of JWFT. After 30 min, equal lipids (containing triglyceride and cholesterol) were given to each group including high-fat diet control group, and then collected the feces excreted of each rat from 0 to 24 h.

The feces were dried under reduced pressure at 40°C. One gram of dry feces was accurately weighed, and extracted with chloroform and methanol (2:1). The fecal TG and TC levels were determined using the kits from Nanjing Jiancheng Bioengineering Institute (Nanjing, China).

### RNA extraction and reverse transcriptase-polymerase chain reaction (RT-PCR)

RNA was extracted from rat liver by homogenization in RNAprep pure tissue kit (TianGen biotech CO. LTD, Beijing). Purity was determined using the 260/280 ratio on a Nanodrop 2000 spectrophotometer (Thermo Scientific, Wilmington, USA). Samples with a 260/280 ratio of 1.90 or greater were used for cDNA synthesis, using a cDNA reverse transcription kit (Takra, 5 × Primescript RT Master Mix) with Bio-Rad S1000™ Thermal cycler, programmed cycle of 37°C for 15 min, 85°C for 5 sec and 4°C until storage at −80°C.

The primers for *SCD1*, *ACC*, *CPT1*, *FAS* and 18S housekeeping gene were referenced the published references [18, 19]. Primers were ordered from Sangon Biotech (Shanghai) Co., Ltd. RT-PCR was performed using SYBR Green qPCR mastermix (Takra, SYBR® Permix Ex Taq™II) on Bio-Rad CFX 96™ real-time PCR system. The reaction volume was 20 μL. The program had an initial incubation of 95°C for 30 sec, followed by 39 cycles of 95°C for 5 sec and 60°C for 30 sec following 39 cycles, a melt curve was produced by increasing the temperature from 60 to 95°C at a rate of 0.5 per minute. Analysis of data was performed by normalizing the threshold cycle number (Δct) of the gene of interest to the Δct for the housekeeping gene, 18S ribosomal RNA, using the 2^-ΔΔct^ method and expression HFC values relative to LJT, MJT, HJT values and expressing in arbitrary units.

### Statistical analysis

Results were expressed as means ± SEM, with the number of determinations (n = 10) representing separate experiment. Data were evaluated at a 0.05 level of significance with one-way ANOVA with post-hoc testing by Fisher’s protected least significant differences procedure.

## Results

### Polyphenol and caffeine contents of JWFT water extracts

As shown in Table 1, the contents of catechins-gallates such as (−)-epigallocatechin gallate (EGCG), (−)-epicatechin gallate (ECG) and (−)-gallocatechin gallate (GCG) were very low in JWFT. Different from ripened pu-erh tea, the content of gallic acid was still very low [16]. These results indicated JWFT’s post-fermentation was not the same as other Chinese dark teas which highly increased the content of gallic acid.

**Table 1:** **The contents of major polyphenols and caffeine of JWFT**

Compound	GA	GC	EGC	C	EC	CA	EGCG	GCG	ECG
Content (mg/g)	0.794	0.804	3.969	ND^*a*^	0.763	22.521	1.966	ND^*a*^	1.904

^*a*^ND, not detected.

GA, gallic acid; GC, (−)-gallocatechin; EGC, (−)-epigallocatechin; C, (+)-catechin; EC, (−)-epicatechin; CA, caffeine; EGCG, (−)-epigallocatechin gallate; GCG, (−)-gallocatechin gallate; ECG, (−)-epicatechin gallate.

### Body weight, abdominal fat weight and serum lipids and letpin levels

**Figure 1:**
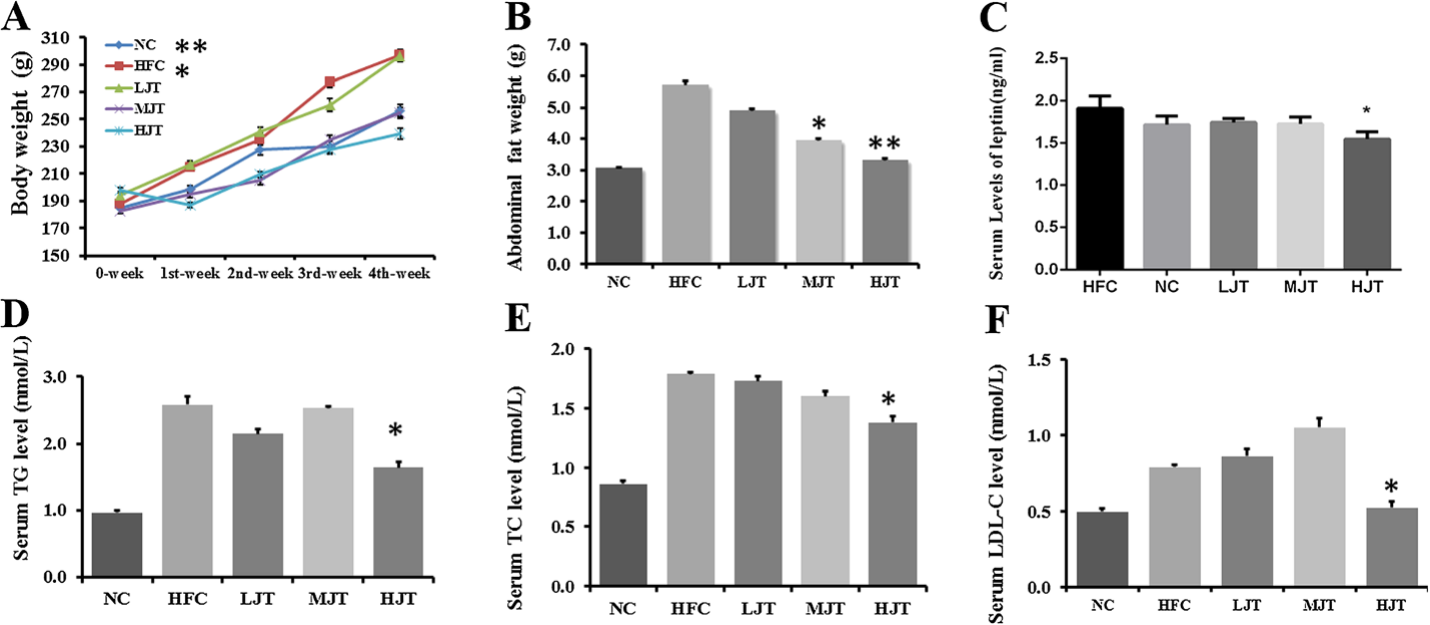
**The effects of JWFT on the lipids levels. (A)** The average body weight of rats each group from 0 to 4 weeks, **(B)** The weights of abdominal fat of rats after 4 weeks, **(C)** The levels of serum leptin of rats after 4 weeks, **(D)** The levels of serum total triglycerides of rats after 4 weeks, **(E)** The levels of serum total cholesterol of rats after 4 weeks, **(F)** The levels of serum LDL-C of rats after 4 weeks. *p < 0.05, **p < 0.01 compared with the HFC group.

The hyperlipidemia rats of HFC group gained more weight during the study than those fed a normal chow diet (p < 0.01). Medium and high dose (1.0, 2.0 g/kg) of JWFT extract exhibited a remarkable decrease in body weight and abdominal fat weight compared with the HFC (Figure 1, p < 0.05, 0.01). Serum TC and TG levels of HFC group were consistently much higher than the NC group after 28 days (p < 0.01). After four weeks’ treatment of high dose of JWFT extract (2.0 g/Kg), serum TC, TG and LDL-C levels of HJT group were significantly decreased compared with HFC group (p < 0.05).

### Hepatic total cholesterol and triglycerides levels

**Figure 2:**
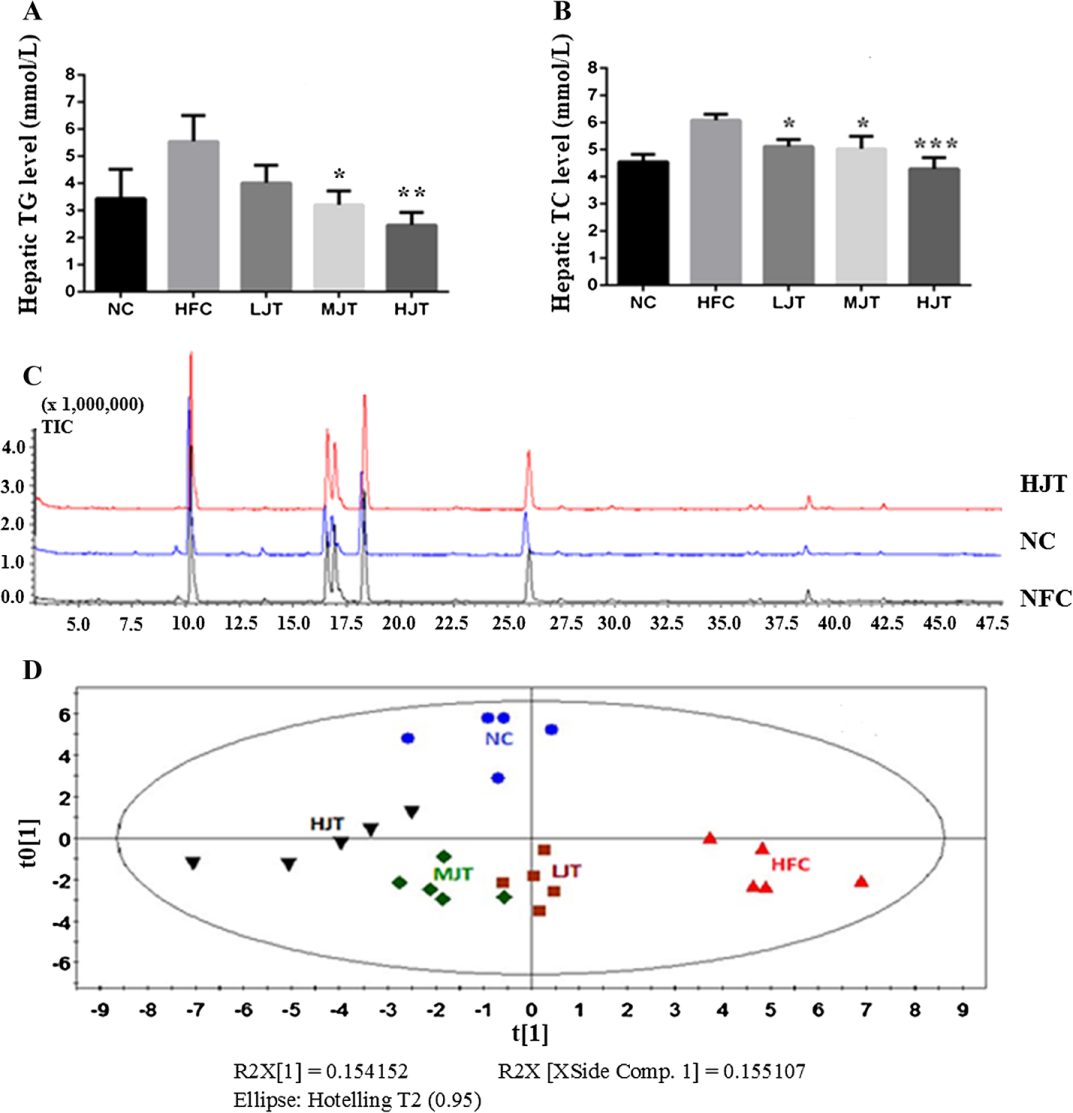
**The GC-MS profiles of lipids of liver. (A)** The levels of total hepatic triglycerides of rats orally administered with JWFT, **(B)** The levels of total hepatic cholesterol of rats orally administered with JWFT, **(C)** The GC-MS of fatty acids of liver of rats in HFC, NC and HJT group, **(D)** The PCA analysis of GC-MS metabolomics of fatty acids. *p < 0.05, **p < 0.01, ***p < 0.001 compared with the HFC group.

High-fat diet caused the elevation of hepatic total TG and TC levels. JWFT treatment decreased the hepatic total TG and TC levels. As shown in Figure 2, hepatic TC levels were dose-dependently decreased by 15.93%, 17.24% and 29.56% at the doses of 0.5, 1.0 and 2.0 g/kg, respectively. JWFT also significantly inhibited the increase in hepatic total TG levels, and exhibited 42.34%, 55.49% reduction at medium and high doses respectively. Although the low dose of JWFT did not reduce TG level evidently, it had 27.57% reduction compared with HFC*.*

The above-mentioned results demonstrated that both medium and high doses of JWFT can lower hepatic total TG and TC levels.

### Analysis of fatty acid and expression of *CPT1*, *ACC*, *SCD1*and *FAS*of liver

Hepatic fatty acid composition was shown in the Table 2. When rat was given high-fat diet, C18:0, C18:1n-9 and C18:2 were significantly increased as previous report [20]. After the treatment of JWFT, the percentages of C14:0, C15:0, C16:1n-7, C17:0, C18:1n-9 were markedly (p < 0.05) decreased, only C18:0 significantly (p < 0.05) elevated in HJT group compared with HFC.The difference of fatty acid composition between HFC and NC group (Figure 2) was profiled systematically by lipidomics. Principal components analysis (PCA) of GC-MS data sets showed that the HFC group was different from NC. JWFT treatment group presented a similar classification to NC group rather than HFC. It suggested that the characteristics of whole fatty acids of JWFT rats were similar to NC rats.

**Table 2:** **The proportional fatty acid composition of liver**

Fatty acid	NC	HFC	LJT	MJT	HJT
C14:0	0.31 ± 0.06	0.215 ± 0.04	0.2 ± 0.02	0.18 ± 0.03	0.1 ± 0.03*
C15:0	0.577 ± 0.06	0.124 ± 0.02	0.094 ± 0.02	0.04 ± 0.02	0.033 ± 0.01*
C16:0	25.17 ± 0.85	24.53 ± 0.49	25.38 ± 0.4	25.59 ± 0.53	23.76 ± 0.65
C16:1n-7	1.83 ± 0.28	0.665 ± 0.12	0.467 ± 0.06	0.429 ± 0.09	0.223 ± 0.05*
C17:0	1.4 ± 0.1	0.393 ± 0.05	0.349 ± 0.02	0.31 ± 0.02	0.241 ± 0.03*
C18:0	17.97 ± 0.67	21.62 ± 0.85	22.38 ± 0.67	23.46 ± 0.59	24.42 ± 0.88*
C18:1n-9	10.8 ± 0.83	15.08 ± 1.01	16.51 ± 1.13	16.14 ± 1.00	12.86 ± 0.73*
C18:1n-7	2.69 ± 0.11	1.75 ± 0.09	1.85 ± 0.34	2.09 ± 0.41	1.52 ± 0.06
C18:2n-6	12.73 ± 0.64	13.02 ± 0.65	13.86 ± 0.24	13.51 ± 0.25	13.43 ± 0.37
C18:3n-3	0.69 ± 0.08	0.76 ± 0.02	0.71 ± 0.05	0.72 ± 0.06	0.67 ± 0.09
C20:3n-3	0.38 ± 0.03	0.27 ± 0.02	0.19 ± 0.01	0.18 ± 0.01	0.23 ± 0.02
C20:3n-6	0.35 ± 0.04	0.18 ± 0.03	0.12 ± 0.02	0.11 ± 0.01	0.16 ± 0.03
C20:4n-6	13.91 ± 0.61	13.30 ± 0.73	12.11 ± 0.45	12.26 ± 0.32	13.41 ± 0.55
C20:5n-3	0.52 ± 0.06	0.32 ± 0.03	0.25 ± 0.02	0.21 ± 0.01	0.26 ± 0.02
C22:6n-3	2.63 ± 0.17	2.34 ± 0.11	1.95 ± 0.15	1.69 ± 0.06	2.70 ± 0.22

NC, normal chow group; HFC, high dose of fat chow group; LJT, low dose of Jing-wei fu tea group; MJT, medium dose of Jing-wei fu tea group; HJT, high dose of Jing-wei fu tea group.

All data are presented as mean ± SEM, *Compared with HFC, *P < 0.05.

**Figure 3:**
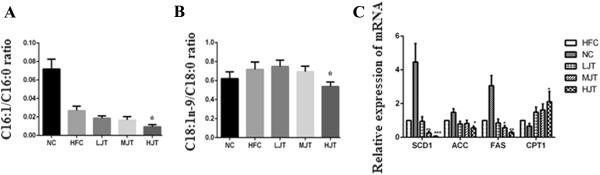
**The effects of JWFT on expression of lipids metabolism genes. (A)**, The ratios of C16:1/C16:0, **(B)** The ratios of C18:1n-9/C18:0, **(C)** The mRNA expression of hepatic lipogenic genes. *P < 0.05, **P < 0.01, ***P < 0.001 compared with the HFC group.

Hepatic stearoyl-CoA desaturase 1 (*SCD1*) activity could be estimated from the C16:1/C16:0 or C18:1n-9/C18:0 ratios. As shown in Figure 3, *SCD1* activity was dose-dependently decreased in JWFT groups, but significantly increased (p < 0.05) in HJT group compared with NC rats. In order to verify the reliability of estimation, we further analyzed the relative expression of *SCD1*, which was evidently (p < 0.05, p < 0.01) down-regulated in MJT and HJT groups with 74%, 96% reduction respectively (Figure 3). Furthermore, the expression of other lipogenic genes, *ACC* and *FAS* were also down-regulated in MJT and HJT groups (p < 0.05, p < 0.01). The expression of *CPT1* was up-regulated in HJT, which indicated the increasing of β-oxidation of fatty acids in liver (p < 0.05).

### Fecal total cholesterol and triglycerides levels

At the 27th day, equivalent lipids (cholesterol and triglycerdies) were given to HFC, LJT, MJT and HJT groups. The feces excreted from 0 to 24th hour were analyzed. In all JWFT treatment groups (0.5, 1.0 and 2.0 g/kg), the total fecal TC levels were highly increased compared with the high fat group (p < 0.05, 0.01) as shown in Table 3.

**Table 3:** **The levels of total cholesterols and total triglycerides of feces**

	HFC	LJT	MJT	HJT
TC (mmol/100 g)	11.14 ± 1.88	17.35 ± 1.32*	18.45 ± 2.56*	18.60 ± 1.29**
TG (mmol/100 g)	6.34 ± 1.00	6.74 ± 0.41	7.48 ± 078	8.63 ± 1.38

HFC, high dose of fat chow group; LJT, low dose of Jing-wei fu tea group; MJT, medium dose of Jing-wei fu tea group; HJT, high dose of Jing-wei fu tea group.

All data are presented as mean ± SEM, *Compared with HFC, *P < 0.05, **P < 0.01.

## Discussion

Dysregulations of hepatic lipid synthesis have been associated with several metabolic diseases. We need a comprehensive understanding of lipogenesis and lipolysis regulation in disease process of metabolic syndrome [21]. For example, nonalcoholic fatty liver disease (NAFLD) is a common disease characterized by excessive accumulation of triglyceride in the liver. Excessive energy intake is an important contributor to the increasing of NAFLD in modern society [22]. In the present study, we intended to investigate the changes related to lipid metabolism of liver tissue induced by high-fat diet using a combined transcriptomic and metabolomic approach [23]. Furthermore, the variations of gene expression indicated the regulation pathways of molecular pathogenesis of hepatic steatosis. In view of the complexity of the hepatic steatosis, our study adopted a strategy of metabolomics profiling for investigating the hepatic fatty acids between normal and hyperlipidemia rats. With PCA analysis of fatty acids composition, it showed that the normal rats were highly different with the high fat induced rats.

After four weeks’ high fat diet feeding, the serum lipids levels, hepatic lipids levels, abdominal fat and body weight were significantly increased. In tea treatment groups, JWFT had the ability to lower the serum lipids (TC, TG and LDL-C). Intra-abdominal fat deposition is a key component of obesity. Some studies indicated that it was an indicator for type 2 diabetes and metabolic syndrome [24]. JWFT had a potent effect on decreasing intra-abdominal fat weight. The results also showed that body weight and abdominal fat weight were positively correlated with serum leptin levels. The serum leptin levels of HFC group was higher than NC group, while the leptin levels of were significantly lowered by JWFT supplementation.

The expression of microRNA of major enzymes involved in de novo synthesis, such as *SCD1*, *ACC* and *FAS* were all significantly decreased after high-fat diet, while the expression of *CPT1* was increased. After the treatment of JWFT, total hepatic cholesterol and triglycerides levels were also decreased markedly by JWFT treatment, which suggested a potentiality on reversing adiposis hepatica. Furthermore, high dose of JWFT can change the fatty acid composition of high-fat fed rats’ liver. Most of saturated and monounsaturated fatty acids percent-age were decreased significantly, such as C14:0, C15:0, C16:1n-7, C17:0 and C18:1n-9. TheC16:1/C16:0 or C18:1n-9/C18:0 ratios may be adjusted by the hepatic *SCD1* activity [20]. The ratio of C16:1/C16:0 and C18:1n-9/C18:0 had a notably reduction with high dose JWFT treatment, meanwhile, the level of relative expression of *SCD1* was down-regulated considerably in MJT and HJT group. So, it was reasonable that the accumulation of *SCD1* substrates (SFA, C16:0 or C18:0) and reduction of products (MUFA, C16:1n-7 or C18:1n-9).

The increased fat accumulation in the liver of HFD rats caused a down-regulation of lipogenic genes and the up-regulation of genes involved in catabolism of free fatty acids, possibly by a feedback mechanism. We found that several enzymes, which have been known to be involved in the de novo synthesis, were down-regulated after the high-fat diet. Parallel down-regulation of *SCD1* in our study at mRNA levels is consistent with potentially reduced fatty acid composition in liver of NAFLD [25]. Furthermore, the *CPT1* was known to be involved in β-oxidation of fatty acid was also up-regulated in the HFLD-fed group. In four weeks’ experiment, the up-regulation of β-oxidation genes and down-regulation of de novo synthesis could be effective on serum lipids if the experiment had been conducted for a longer duration.

## Conclusions

In conclusion, JWFT exhibited a potential anti-obesity effects for high-fat diet induced mice, which may be mediated by firstly inhibiting the absorption of lipids, secondly strengthening the feedback regulation of expression of de novo lipogenic genes, and up-regulating *CPT1* expression in the liver. Furthermore, the RT-PCR results also showed that rats resisted the over uptake of lipids by down-regulating the expression of lipogenic genes. It was supposed that in a short treatment, the absorption of lipids was firstly controlled, because the low dose of JWFT also promoted the excretion of lipid in feces. In a long term, the regulation of metabolism should be responsible for the lipids control.

## Abbreviations

CDT: Chinese dark tea
JWFT: Jingwei fu tea
GA: Gallic acid
C: (+)-Catechin
EC: (−)-Epicatechin
GC: (−)-Gallocatechin
EGC: (−)-Epigallocatechin
GCG: (−)-Gallocatechin gallate
EGCG: (−)-Epigallocatechin gallate
ECG: (−)-Epicatechin gallate
TC: Total cholesterol
TG: Total triglycerides
NC: Normal chow group
HFC: High dose of fat chow group
LJT: Low dose of Jing-wei fu tea group
MJT: Medium dose of Jing-wei fu tea group
HJT: High dose of Jing-wei fu tea group
SCD1: Stearoyl-CoA desaturase 1
ACC: Acetyl-CoA carboxylase
FAS: Fatty acid synthase
MUFAs: Monounsaturated fatty acids
CPT1: Carnitine palmitoyltransferase 1.

## References

[1] Balthasar N, Dalgaard LT, Lee CE, Yu J, Funahashi H, Williams T, Ferreira M, Tang V, McGovern RA, Kenny CD, Christiansen LM, Edelstein E, Choi B, Boss O, Aschkenasi C, Zhang CY, Mountjoy K, Kishi T, Elmquist JK, Lowell BB (2005). Divergence of melanocortin pathways in the control of food intake and energy expenditure. Cell.

[2] Schwarz JM, Linfoot P, Dare D, Aghajanian K (2003). Hepatic de novo lipogenesis in normoinsulinemic and hyperinsulinemic subjects consuming high-fat, low-carbohydrate and low-fat, high-carbohydrate isoenergetic diets. Am J Clin Nutr.

[3] Strable MS, Ntambi JM (2010). Genetic control of de novo lipogenesis: role in diet-induced obesity. Crit Rev Biochem Mol Biol.

[4] Klaus S, Pultz S, Thone-Reineke C, Wolfram S (2005). Epigallocatechin gallate attenuates diet-induced obesity in mice by decreasing energy absorption and increasing fat oxidation. Int J Obes (Lond).

[5] Stefanovic-Racic M, Perdomo G, Mantell BS, Sipula IJ, Brown NF, O’Doherty RM (2008). A moderate increase in carnitine palmitoyltransferase 1a activity is sufficient to substantially reduce hepatic triglyceride levels. Am J Physiol Endocrinol Metab.

[6] Ntambi JM, Miyazaki M (2004). Regulation of stearoyl-CoA desaturases and role in metabolism. Prog Lipid Res.

[7] Kumar S, Pandey A (2012). Antioxidant, lipo-protective and antibacterial activities of phytoconstituents present in Solanum xanthocarpum root. Int Rev Biophysical Chem.

[8] Mishra A, Kumar S, Bhargava A, Sharma B, Pandey AK (2011). Studies on in vitro antioxidant and antistaphylococcal activities of some important medicinal plants. Cell Mol Biol.

[9] Yuan JM, Sun C, Butler LM (2011). Tea and cancer prevention: epidemiological studies. Pharmacol Res.

[10] Uchiyama S, Taniguchi Y, Saka A, Yoshida A, Yajima H (2011). Prevention of diet-induced obesity by dietary black tea polyphenols extract in vitro and in vivo. Nutrition.

[11] Hou Y, Shao W, Xiao R, Xu K, Ma Z, Johnstone BH, Du Y (2009). Pu-erh tea aqueous extracts lower atherosclerotic risk factors in a rat hyperlipidemia model. Exp Gerontol.

[12] Wang S, Noh SK, Koo SI (2006). Epigallocatechin gallate and caffeine differentially inhibit the intestinal absorption of cholesterol and fat in ovariectomized rats. J Nutr.

[13] Wang W, Zhang L, Wang S, Shi S, Jiang Y, Li N, Tu P (2014). 8-C N-ethyl-2-pyrrolidinone substituted flavan-3-ols as the marker compounds of Chinese dark teas formed in the post-fermentation process provide significant antioxidative activity. Food Chem.

[14] Li Q, Liu Z, Huang J, Luo G, Liang Q, Wang D, Ye X, Wu C, Wang L, Hu J (2013). Anti-obesity and hypolipidemic effects of Fuzhuan brick tea water extract in high-fat diet-induced obese rats. J Sci Food Agric.

[15] Xu A, Wang Y, Wen J, Liu P, Liu Z, Li Z (2011). Fungal community associated with fermentation and storage of Fuzhuan brick-tea. Int J Food Microbiol.

[16] Zhang L, Li N, Ma Z-Z, Tu P-F (2011). Comparison of the chemical constituents of aged pu-erh tea, ripened pu-erh tea, and other teas using HPLC-DAD-ESI-MSn. J Agric Food Chem.

[17] Folch J, Lees M, Sloane Stanley GH (1957). A simple method for the isolation and purification of total lipides from animal tissues. J Biol Chem.

[18] Marks KA, Kitson AP, Stark KD (2013). Hepatic and plasma sex differences in saturated and monounsaturated fatty acids are associated with differences in expression of elongase 6, but not stearoyl-CoA desaturase in Sprague–Dawley rats. Genes Nutr.

[19] Suchankova G, Tekle M, Saha AK, Ruderman NB, Clarke SD, Gettys TW (2005). Dietary polyunsaturated fatty acids enhance hepatic AMP-activated protein kinase activity in rats. Biochem Biophys Res Commun.

[20] Kim HJ, Kim JH, Noh S, Hur HJ, Sung MJ, Hwang JT, Park JH, Yang HJ, Kim MS, Kwon DY, Yoon SH (2011). Metabolomic analysis of livers and serum from high-fat diet induced obese mice. J Proteome Res.

[21] Avramoglu RK, Basciano H, Adeli K (2006). Lipid and lipoprotein dysregulation in insulin resistant states. Clin Chim Acta.

[22] Milagro FI, Campion J, Martinez JA (2006). Weight gain induced by high-fat feeding involves increased liver oxidative stress. Obesity (Silver Spring).

[23] Xie Z, Li H, Wang K, Lin J, Wang Q, Zhao G, Jia W, Zhang Q (2010). Analysis of transcriptome and metabolome profiles alterations in fatty liver induced by high-fat diet in rat. Metabolism.

[24] Despres JP (2006). Intra-abdominal obesity: an untreated risk factor for Type 2 diabetes and cardiovascular disease. J Endocrinol Invest.

[25] Musso G, Gambino R, Cassader M (2009). Recent insights into hepatic lipid metabolism in non-alcoholic fatty liver disease (NAFLD). Prog Lipid Res.

[CR26] The pre-publication history for this paper can be accessed here: http://www.biomedcentral.com/1472-6882/14/263/prepub

